# Remodeling Cildb, a popular database for cilia and links for ciliopathies

**DOI:** 10.1186/2046-2530-4-S1-P21

**Published:** 2015-07-13

**Authors:** O Arnaiz, J Cohen, AM Tassin, F Koll

**Affiliations:** 1 Centre de Génétique Moléculaire, CNRS, Gif Sur Yvette, France

## 

Cildb (http://cildb.cgm.cnrs-gif.fr/) is a multi-species knowledgebase gathering high throughput studies, which allows advanced searches to identify proteins involved in centrosome, basal body or cilia biogenesis, composition and function. Combined to localization of genetic diseases on human chromosomes given by OMIM links, candidate ciliopathy proteins can be compiled through Cildb searches. Since its creation in 2009, Cildb has been updated twice and the latter version displays a novel BioMart interface, much more intuitive than the previous ones. We describe here the novelties of the new version V3.0 and give an example on advanced search on centrosomal proteins to illustrate the evolutionary conservation viewed through Cildb.

